# Evaluation of anti-inflammatory and immunomodulatory potential of Lawsone (2-hydroxy-1,4-naphthoquinone) using pre-clinical rodent model of rheumatoid arthritis

**DOI:** 10.3389/fphar.2023.1279215

**Published:** 2023-10-13

**Authors:** Sara Sattar, Arham Shabbir, Muhammad Shahzad, Tasleem Akhtar, Syed Muneeb Anjum, Mohammed Bourhia, Hiba-Allah Nafidi, Yousef A. Bin Jardan, Musaab Dauelbait, Aisha Mobashar

**Affiliations:** ^1^ Department of Pharmacology, Faculty of Pharmacy, The University of Lahore, Lahore Campus, Lahore, Pakistan; ^2^ Department of Pharmacology, Institute of Pharmacy, Faculty of Pharmaceutical and Allied Health Sciences, Lahore College for Women University, Lahore, Pakistan; ^3^ Department of Pharmacology, University of Health Sciences, Lahore, Pakistan; ^4^ Institute of Pharmaceutical Sciences, University of Veterinary and Animal Sciences, Lahore, Pakistan; ^5^ Department of Chemistry and Biochemistry, Faculty of Medicine and Pharmacy, Ibn Zohr University, Laayoune, Morocco; ^6^ Department of Food Science, Faculty of Agricultural and Food Sciences, Laval University, Quebec, QC, Canada; ^7^ Department of Pharmaceutics, College of Pharmacy, King Saud University, Riyadh, Saudi Arabia; ^8^ Department of Scientific Translation, Faculty of Translation, University of Bahri, Khartoum, Sudan

**Keywords:** arthritis, inflammation, cytokines, rheumatism, phytochemical

## Abstract

**Background:** Lawsone (2-hydroxy-1,4-naphthoquinone) is naturally present in *Lawsonia Inermis* and flowers of *Eicchornia crassipes*. This study assessed the anti-arthritic potential of Lawsone, using FCA-induced Sprague-Dawley rats.

**Methods:** Arthritic progress was analyzed through a macroscopic scoring scale, measurement of paw edema, and histopathological changes. Effects of Lawsone on mRNA expression levels of inflammatory markers were examined using the reverse transcription PCR technique. ELISA technique was used to evaluate the PGE2 levels. Moreover, levels of biochemical and hematological parameters were also analyzed.

**Results:** The research elucidated that Lawsone showed an inhibitory potential towards arthritic progress and ameliorated the paw edema. The histopathological analysis also validated the inhibition in arthritic development. Treatment with Lawosne reduced the expression levels of inflammatory markers in rats i.e., VEGF, TNF-α, MMP-2, MMP-3, NF-κB, IL-1β, and IL-6. PGE2 levels (all *p* < 0.001) were also found reduced in treatment groups. Lab investigations showed improved results of hematological and hepatic parameters in the treated groups as compared to the positive control. This study found no hepatotoxic or nephrotoxic effects of Lawsone in the test doses.

**Conclusion:** Lawsone possesses an anti-arthritic property which could be ascribed to its immunomodulatory and anti-inflammatory effects.

## 1 Introduction

RA is defined as an autoimmune multisystem disease, having primary symptoms like pain, inflammation of synovial membrane & peripheral joints, deterioration of articular tissues, morning stiffness, and constrained joint mobility (Scherer, Haupl, and Burmester, 2020).

It involves multiple systems and has a complex pathology (Kaur and Sultana, 2012). RA involves both immune cells, i.e., B-cells, T-cells, macrophages, and soluble inflammatory mediators of our defense system (Yap et al., 2018). RA development is the consequence of the imbalance between the expressed values of pro-inflammatory and anti-inflammatory cytokines, resulting in the proliferation of cells in the synovial layer and infiltration of cytokines, chemokines, growth factors, and hormones in the joints RA (Bugatti et al., 2014). RA can also result in severe disabilities, hence compromising the quality of life in both physical and mental spheres (Matcham et al., 2014).

In the case of RA, the average mortality ratio varies from 1.28 to 2.89. The overall data from the population of developed countries shows the RA occurrence rate to be 0.5%–1% (Uroos et al., 2017; Li and Zhang, 2020). As per World Health Organization (WHO) report, RA causes 0.8% of total global years of life lost due to disability (YLD). According to the Global Burden of Disease (GBD) 2017 report, the prevalence of RA is higher in developed countries, such as North America, Western Europe, and the Caribbean, followed by India and South America (Finckh et al., 2022). The risk of RA seems to be greater in women than men and also enhances with age (Shabbir et al., 2014). The prominent risk factors for RA are female gender, age, tobacco use, silica exposure, and obesity (Yap et al., 2018).

Inflammatory and edematous conditions like RA are usually treated by corticosteroids, non-steroidal anti-inflammatory drugs (NSAIDs), disease-modifying anti-rheumatic drugs (DMARDs), opiates and anti-TNFs. NSAIDs, like piroxicam, are preferred to be the “first-line” therapeutic drugs for the treatment of RA, as they restrict the upregulation of IL-6, IL-1, TNF-α, and pro-inflammatory prostaglandins by blocking the cyclooxygenase pathway of the arachidonic acid cascade. DMARDs, e.g., methotrexate, are immune-modulators that regulate a disturbed immune system (Uroos et al., 2017). American College of Rheumatology guidelines for the treatment of RA (2021) strongly recommends the use of methotrexate as monotherapy with moderate-to-high RA disease activity for DMARD naïve patients. These recommendations are over the use of sulfasalazine or hydroxychloroquine. The guidelines also acknowledge that short term glucocorticoids are often important to reduce the symptoms of RA before the beginning of onset of action of DMARDs. However, the duration of glucocorticoid therapy should be as short as possible and the lowest effective dose should be used. Along with this acknowledgment, the guidelines conditionally recommend the use of conventional synthetic DMARDs without short term glucocorticoids (Fraenkel et al., 2021).

DMARDs cause mild alopecia, rash, nausea, loss of appetite, raised rheumatoid nodules, oral ulcers and other neurological complications (Guo et al., 2018). Anti-TNFs drugs inhibit the expression of TNF and block the inflammatory response. But they cause headache, abdominal pain, vomiting, diarrhea, itching, bruising, bleeding, rash, cellulitis, and lower respiratory tract complications. Corticosteroids such as prednisone hormones, effectively reduce synovitis. However, long-term usage of corticosteroids also includes increase risk of osteoporosis, diabetes mellitus, peptic ulcer, gastrointestinal bleeding, cataracts, and infections. Short-term opiates, such as fentanyl and oxycodone**,** help in pain management in RA but the long-term ones are linked with side effects such as respiratory depression, dependence, and tolerance (Mobashar et al., 2020a).

All of this demands the development of a novel but safe anti-arthritic regimen (Uroos et al., 2017). There have been discovered many plant-based products that showed significant effects in the reduction of chronic joint inflammation, such as rheumatoid arthritis (Bang et al., 2009; Mubashir et al., 2014).

Lawsone (Lawsonia, 2-hydroxy-1,4 napthaquinone), has the orange-red colour artifact and is naturally present in *Lawsonia Inermis* and flowers of *Eicchornia crassipes* (Kapadia et al., 2013). It has anti-inflammatory, hepatoprotective, anti-bacterial, anti-fungal, molluscicidal, anti-parasitic, and antiplatelet and anti-cancer activities (Cape, Bowman, and Kramer, 2006; Adeli-Sardou et al., 2019). Its wound-healing activity was reported by using ethanolic extract of Lawsone which depicted a visible wound-healing effect in incision and excision models of rat. Lawsone increased the pentobarbitone-induced sleeping time in an anti-inflammatory model of rats. It is also used in folk medicine for treatment of inflammatory diseases (Badoni Semwal et al., 2014). The *in vitro* anti-inflammatory potential of the Lawsone complexes depicted the modulation in the activity of NF-κB and TNF-α with results similar to prednisolone (Vanco et al., 2017). It also proved to be effective the in amelioration of interleukins levels, resulting in the reduction of inflammation and edema (Biradar and Veeresh, 2013). A study has previously reported cytotoxic effects of lawsone which are probably mediated through the release of O_2_^−^, H_2_O_2_, and OH^−^. The study indicated that Lawsone is non-mutagenic (Sauriasari et al., 2007). This research assessed the anti-arthritic potential of Lawsone, using FCA-induced Sprague-Dawley rats.

## 2 Methodology

### 2.1 Test animals

Sprague-Dawley rats of both sexes, with a weight range of 150–250 g, were subjected to investigate the anti-arthritic property of Lawsone. Sprague-Dawley rats were obtained from the University of Veterinary & Animal Sciences, Lahore. Rats were fed with a standard pellet diet and water *ad libitum*. They were kept under 12 h dark/light cycles and average conditions of humidity (60%–70%) and temperature of (28°C ± 2°C) at the University of Veterinary & Animal Sciences, Lahore. They were familiarized with the surroundings 1 week earlier than the trial (Kyei, Koffuor, and Boampong, 2012).

### 2.2 Assessment of anti-arthritic effect

30 Sprague-Dawley rats were dispersed into 5 groups. Except for the negative control (group 1), arthritis was induced in the rest of the 4 groups by injecting FCA (0.1 mL) into the sub-plantar area, starting from day 0. Group 1 and arthritic control, i.e., group 2 were fed with normal saline (NS) per oral (Akhtar and Arham Shabbir, 2019), from day 8–22. The reference group (group 3) was dosed with piroxicam (10 mg/kg b.w., i.p.) (Shabbir et al., 2014). Group 4 was given Lawsone (100 mg/kg b.w., p.o.) and group 5 was given Lawsone (200 mg/kg b.w., p.o.) from day 8–22. The anti-inflammatory doses of Lawsone were selected from the literature review. Previously Lawsone has shown anti-inflammatory and anti-oxidant activity in in vivo rat models of inflammation at the doses of 100 and 200 mg/kg b.w. (Biradar and Veeresh, 2013). Lawsone was obtained from Alfa Aesar (LOT: 10192582).

### 2.3 Measurement of the arthritic score and paw edema

Morphological features of arthritis were analyzed macroscopically for all the rats on the 8^th^, 13th, 18th, and 23rd days (Akhtar and Arham Shabbir, 2019). A score of 0 was used for the normal paw. Scores 1 to 2 were for mild to moderate inflammation and erythema of digits. Score 3 was given to severe inflammation and erythema of digits. Score 4 was given to significant deformity and incapability of movement of limbs. The paw volume was also recorded on the same days using a digital vernier caliper.

### 2.4 Histopathological evaluation

The ankle joints of all groups, injected with FCA, were separated and fixed with 10% formalin, and afterward decalcified by a decalcifying agent (10% formic acid with 10% formalin). Then, samples were fixed with paraffin and stained by the H&E method, after cutting into slices of 5 µm thickness. Arthritic parameters such as bone eruption, formation of pannus, and intrusion of inflammatory cells were analyzed. Results were predicted through a scale having 0, 1, 2, 3, and 4 ranges for normal, mild, moderate, and gross variations respectively.

### 2.5 Analysis of mRNA expression levels of pro-inflammatory cytokines: TNF-α, IL-1β, IL-6, NF-κB, MMP-2, MMP-3, and VEGF

Blood samples of rats were collected and RNA is extracted using the Total RNA Isolation (TRIzol) method. A Nanodrop spectrophotometer (major science mini-300) was used to quantify the total RNA extracted. Then, cDNA was generated by following the RevertAid First Strand cDNA synthesis kit’s protocol (Thermo Scientific LOT 00960732). GAPDH was taken as a housekeeping gene. Primers of IL-1β, MMP-2, and MMP-3 were designed manually. Sequences of other primers, i.e., IL-6, TNF-α, NF-κB, and VEGF were followed from previously published papers, as exhibited in Table 1. cDNA (1 µL) was centrifuged with forward-reverse primer mix (1 µL), nuclease-free water, and PCR Master Mix (5 µL). The thermal cycler was operated for 35 cycles of the following 3 phases: denaturation (95°C for 10 s), annealing (according to temperatures displayed in Table 4) for 20 s, and subsequently extension (72°C for 30 s) cycles.

**TABLE 1:** Primer sequences.

Genes	Forward primer	Reverse primer	Annealing temperature (°C)	Product size	Reference
IL-1β	5′-CCT​GCT​AGT​GTG​TGA​TGT​TC-3′	5′-GAG​GTG​CTG​ATG​TAC​CAG​TT-3′	58	390	ENSRNOG0000004649
IL-6	5′-AGA​CTT​CCA​GCC​AGT​TGC​CT-3′	5′-CTG​ACA​GTG​CAT​CAT​CGC​TG-3′	60	233	(Uttra et al., 2018)
TNF-α	5′-CCT​CTT​CTC​ATT​CCT​GCT​CGT-3″	5′-TGA​GAT​CCA​TGC​CAT​TGG​CC-3′	60	266	(Shabbir et al., 2016)
NF-κB	5′-CAA​GGA​AGA​GGA​TGT​GGG​GTT-3	5′-AGC​TGA​GCA​TGA​AGG​TGG​ATG-3′	60	207	(Shabbir et al., 2016)
VEGF	5′-GTT​CAG​AGC​GGA​GAA​AGC​ATT-3	5′-CTT​GCA​ACG​CGA​GTC​TGT​GT-3′	60	80	(Zhu et al., 2019)
MMP-2	5′-CGA​ACA​AGT​ATG​AGA​GCT​GC-3′	5′-CGG​TCA​TCA​TCG​TAG​TTG​GT-3′	57	85	ENSRNOG00000016695
MMP-3	5′-CCT​TTT​GAT​GGG​CCT​GGA​AT-3′	5′-GTG​ACA​TCA​TCT​GTC​CAT​CG-3′	58	107	ENSRNOG00000032626
GAPDH	5′-GTC​ATC​AAC​GGG​AAA​CCC​AT-3′	5′-CTT​GCC​GTG​GGT​AGA​GTC​AT-3	60	229	(Shabbir et al., 2016)

### 2.6 Determination PGE2 levels

Rat Prostaglandin E2 ELISA kit (Bioassay Technology Laboratory Cat No. E-0504Ra) was used to determine serum levels of PGE2. Results were obtained by measuring Optical Density (OD) using ELISA reader (BIORAD, PR 4100) at 450 nm wavelength.

### 2.7 Assessment of hematological parameters

An automated hemocytometer was used to calculate the hemoglobin (Hb) content as well as WBCs, RBCs, and platelet counts.

### 2.8 Biochemical parameters

Serum samples were processed to calculate the values of AST, ALT, urea and creatinine, through an automated chemistry analyzer.

### 2.9 Statistical analysis

Data were given as Mean ± SD, using one-way analysis of variance (ANOVA) and *post hoc* Tukey’s test, to arbitrate the significant difference among groups. Here, *p* < 0.05 was reflected as statistically significant.

## 3 Results

### 3.1 Lawsone repressed arthritic progress

**FIGURE 1:**
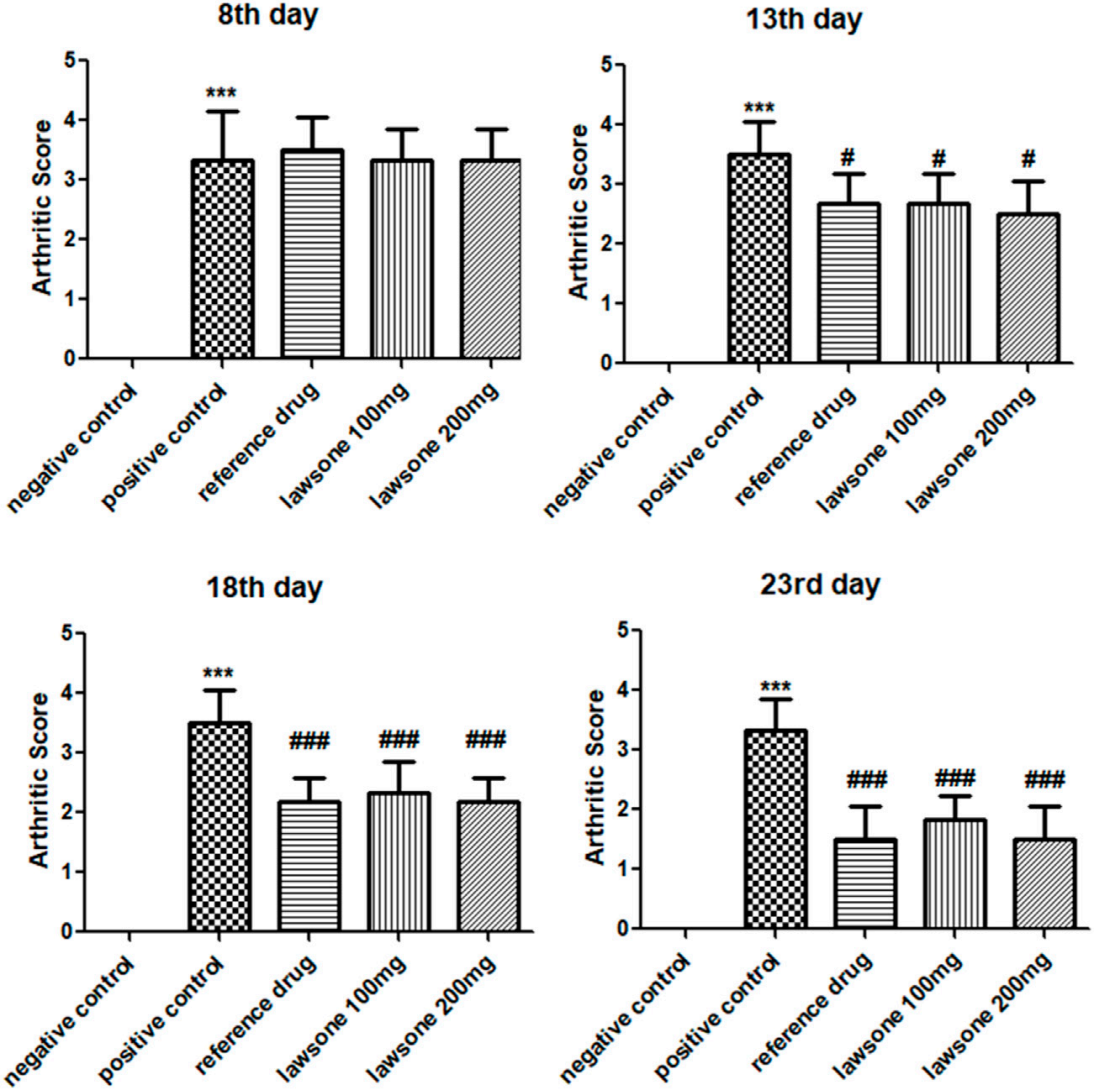
showing arthritic score in all groups after arthritic induction by FCA on 8^th^, 13th, 18th & 23rd day. Data is calculated as mean ± SD (*n* = 06). ***p < 0.001 in comparison to vehicle control group and #*p* < 0.05; ##*p* < 0.01; ###*p* < 0.001 in correlation to arthritic control group. Values of negative control group are zero, therefore are non-visible.

Arthritic development, manifesting swelling and erythema, was observed macroscopically after sub-planter induction of FCA. Treatment with Lawsone was initiated from day 8^th^ till the 23rd day. The arthritic control group indicated a significant (*p* < 0.001) escalation in arthritic development than the vehicle control group since the 8^th^ day. Lawsone treated groups displayed significant diminution in arthritis in contrast to group 2 on the 13th, 18th, and 23rd day respectively. On the 18th and 23rd days, macroscopic observation depicted a significant decrease in arthritis-related swelling and erythema in groups treated with Lawsone as shown in Figure 1.

### 3.2 Lawsone decreased paw edema

**FIGURE 2:**
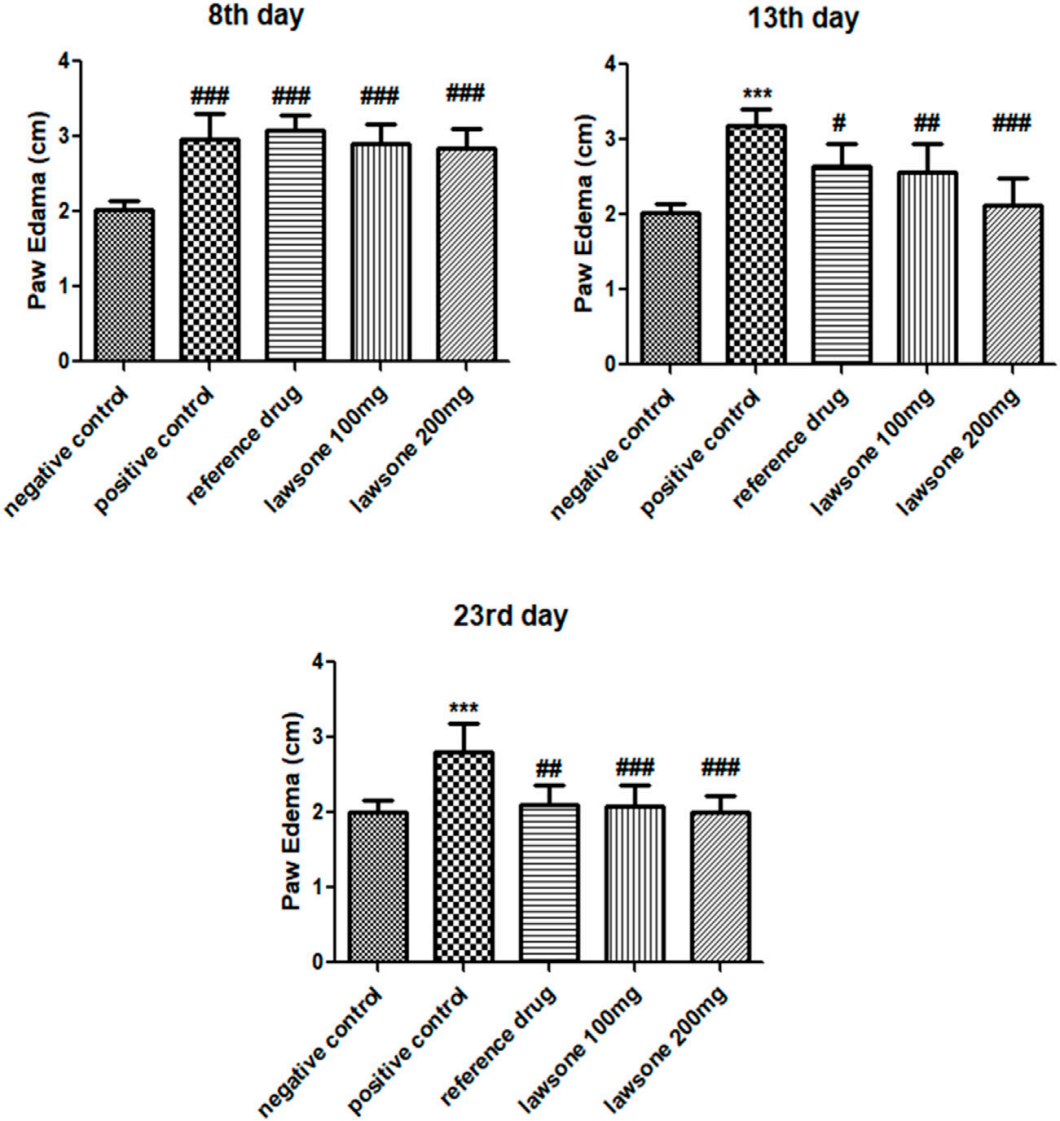
On 8^th^ day, treatment groups were compared with negative control group, while on other days, they were compared with positive control. Data is calculated as mean ± SD (*n* = 06). ***p < 0.001 in correlation to vehicle control group and #*p* < 0.05; ##*p* < 0.01; ###*p* < 0.001 in correlation to arthritic control group.

After FCA induction, paw edema was measured in all groups of Sprague Dawley rats using a digital vernier caliper on the 8^th^, 13th, and 23rd days. Paw volumes observed on the 8^th^ day exhibited a significant rise in paw edema than the vehicle control group. On the other hand, Lawsone treated groups displayed a significant decline in paw edema on the 13th day than the positive control group. On, the 23rd day, all treated groups exhibited significantly reduced levels of paw edema (*p* < 0.001) in contrast to the positive control group as presented in Figure 2.

### 3.3 Lawsone modulated histopathological parameters

**FIGURE 3:**
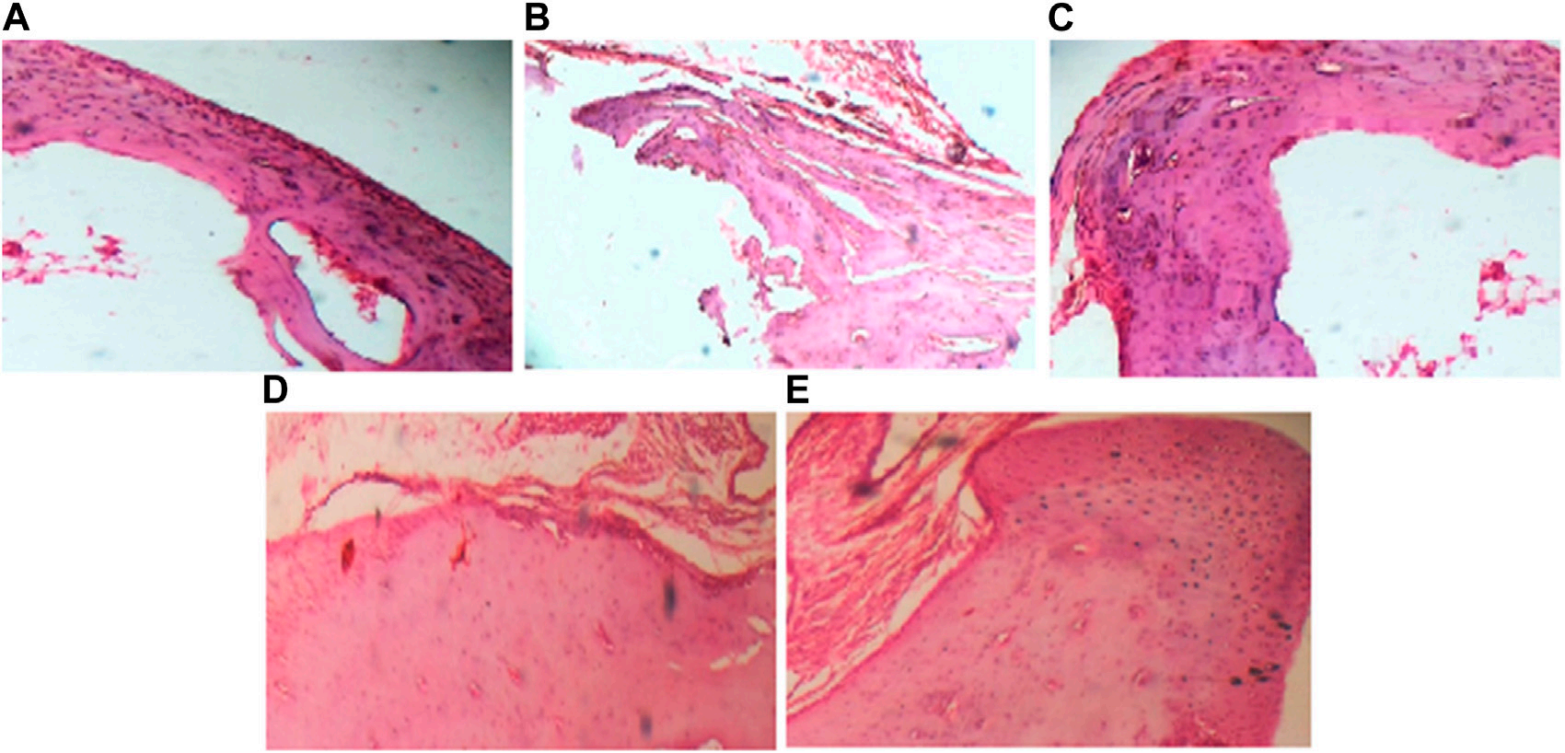
(40x) Histopathological evaluation of Sprague Dawley rats after FCA induction and treatment. **(A)** depicts negative control group; **(B)** depicts positive control group; **(C)** depicts reference drug group; **(D)** depicts group treated with Lawsone 100 mg; **(E)** depicts group treated with Lawsone 200 mg.

**FIGURE 4:**
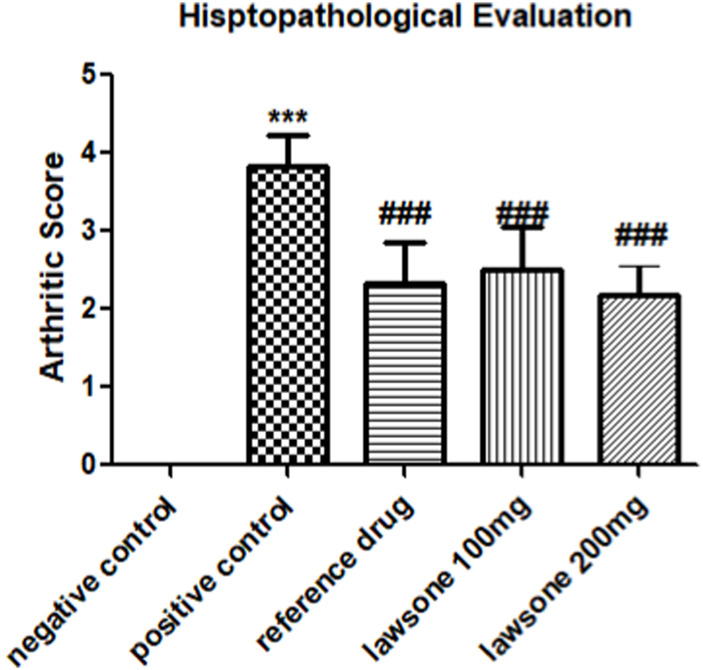
Histopathological evaluation of Sprague Dawley rats after FCA induction and treatment. Data is calculated as mean ± SD (*n* = 06). ***p < 0.001 as compared to vehicle control group and ###*p* < 0.001 in contrast to arthritic control group.

Histopathological evaluation showed a significant reduction in arthritic score in the arthritic control group than the vehicle control group. All treated groups demonstrated attenuation of arthritic score at day 23 as shown in Figure 3 and Figure 4.

### 3.4 Lawsone declined the mRNA expression values of TNF-α, IL-1β, IL-6 & NF-κB

**FIGURE 5:**
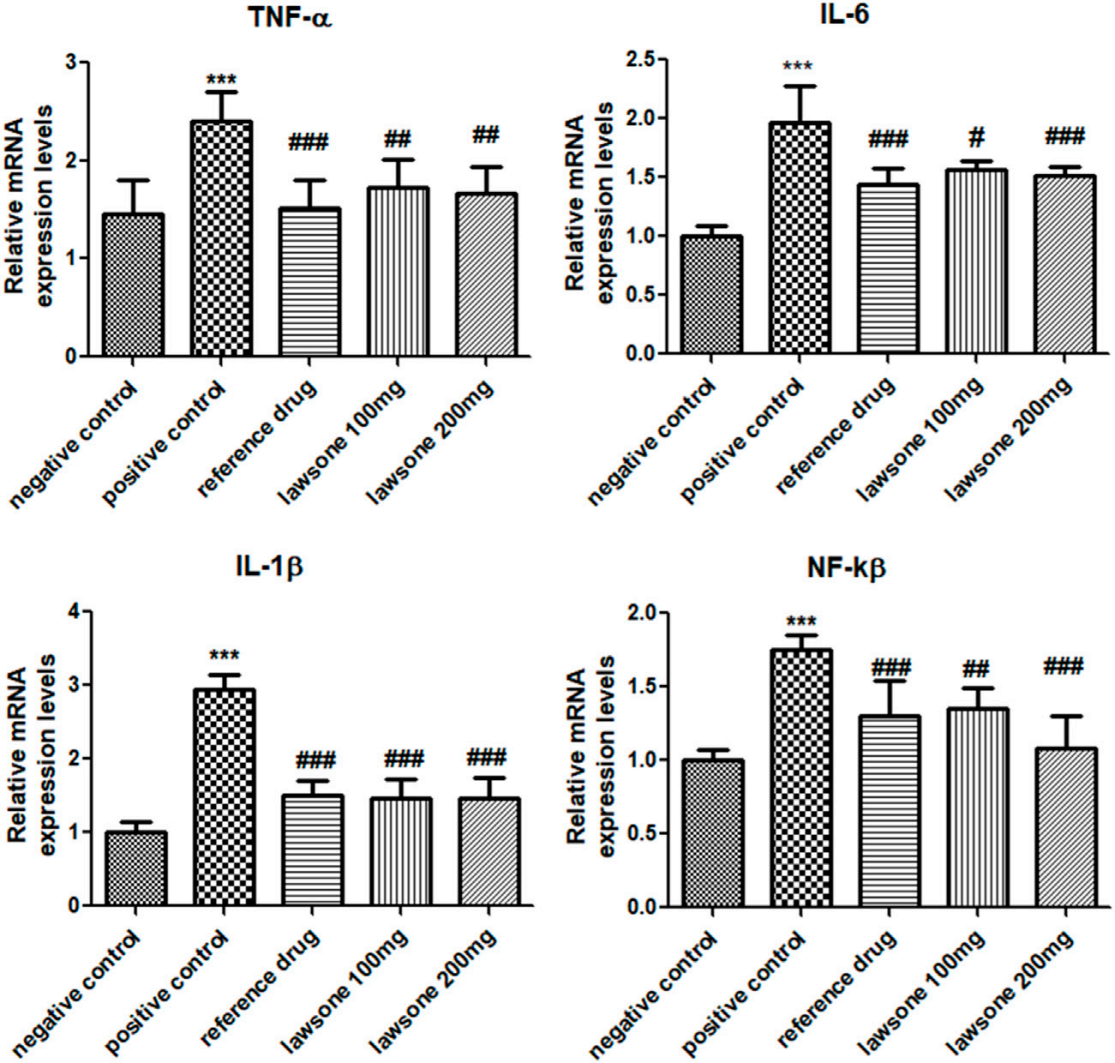
Lawsone reduced the mRNA expression values of TNF-α, IL-1β, IL-6 and NF-κB. Values are stated as mean ± SD (*n* = 06). ***p < 0.001 in contrast to vehicle control group and #*p* < 0.05; ###*p* < 0.001 in correlation to arthritic control group.

Blood samples treated with Lawsone were collected and processed. Significant downregulation of pro-inflammatory cytokines and matrix metalloproteinase enzymes was found, as shown in Figure 5. Arthritic control showed significant upregulation of TNF-α, IL-1β, and IL-6 in comparison to the vehicle control group. Expression levels of all these markers were significantly suppressed by the phytochemical as compared to the arthritic control group.

The positive control group displayed a significant (*p* < 0.001) escalation of NF-κB than the vehicle control group. Significantly (*p* < 0.001) reduced expressed values of NF-κB were found in Lawsone-treated groups than the arthritic control group.

### 3.5 Lawsone attenuated MMP-2, MMP-3 and VEGF expression levels

**FIGURE 6:**
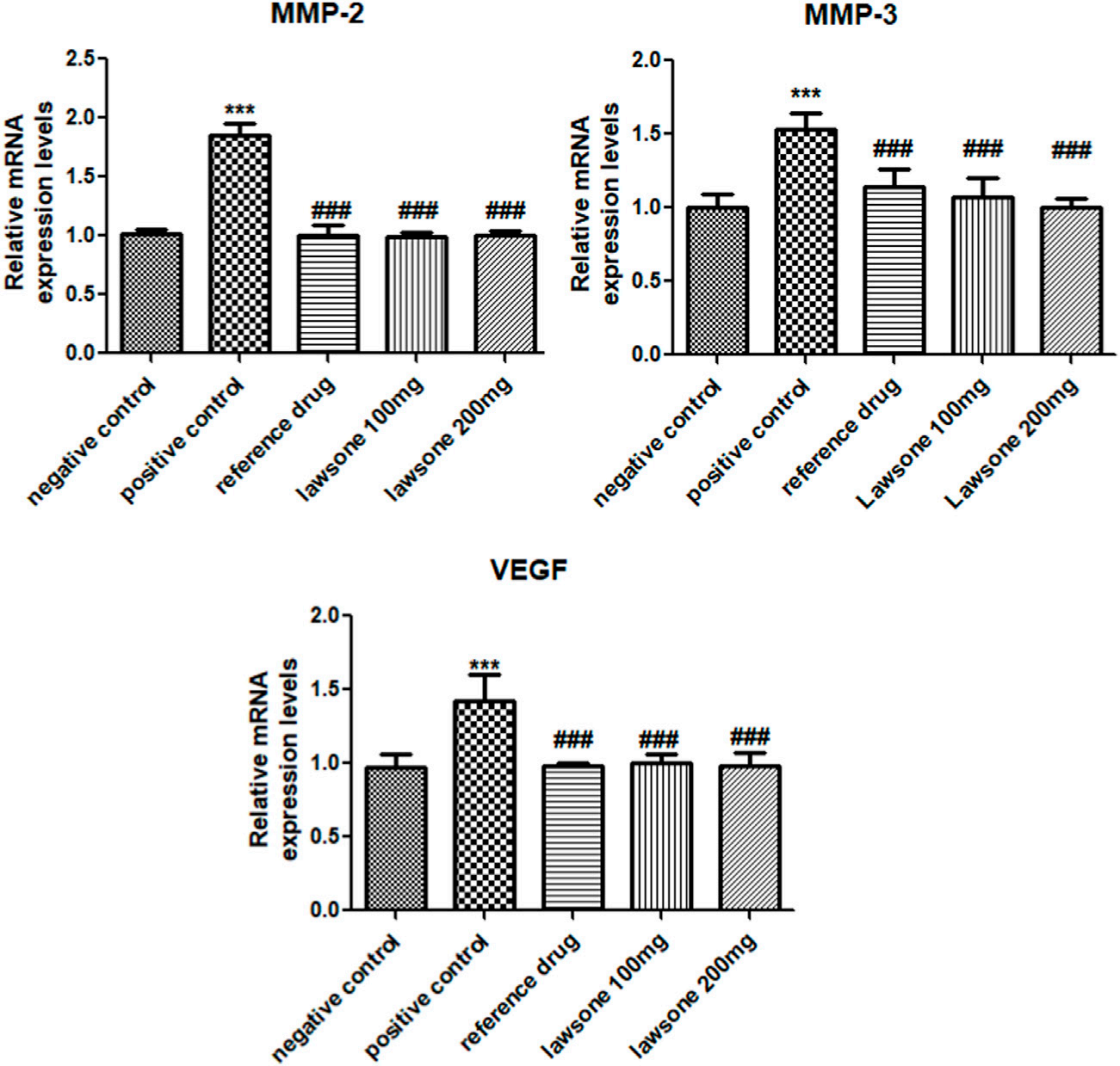
Lawsone declined the mRNA expression values of MMP-2, MMP-3 & VEGF. Values are calculated as mean ± SD (*n* = 06). ***p < 0.001 in contrast to vehicle control group and #*p* < 0.05; ##*p* < 0.001; ###*p* < 0.001 in contrast to arthritic control group.

Significant (*p* < 0.001) upregulation of MMP-3, MMP-2 & VEGF in the arthritic control group was observed as opposed to the vehicle control group. However, suppression of MMP-3, MMP-2, and VEGF levels was found statistically significant after treatment with Lawsone than the positive control group as shown in Figure 6.

### 3.6 Lawsone significantly reduced PGE2 levels

A substantial (*p* < 0.001) raise of PGE2 was depicted in the positive control group as compared to the negative control group. However, when groups treated with Lawsone showed a significant (*p* < 0.001) decline of PGE2 levels than the arthritic control group as shown in Table 2.

**TABLE 2:** Lawsone significantly declined PGE2 Levels as compared to arthritic control group. Data is stated as mean ± SD (n = 06). ***p < 0.001 in contrast to vehicle control and ###*p* < 0.001 as compared to arthritic control group.

Groups	PGE2 (pg/mL)
Vehicle control	0.445 ± 0.023
Arthritic control	4.598 ± 0.65***
Reference drug	1.061 ± 0.161**###**
Lawsone 100 mg	1.052 ± 0.192**###**
Lawsone 200 mg	0.874 ± 0.061**###**

### 3.7 Lawsone modulated hematological markers

A significant decline (*p* < 0.001) in RBC and Hb content was detected in the arthritic control group than in the vehicle control group. Lawsone treated groups showed an increase in RBC and Hb content to the positive control group. The data showed non-significant difference among all groups for TLC count.

However, the arthritic control group presented a significant elevation (*p* < 0.001) in PLT count than the vehicle control group. Lawsone treated groups showed lessened values (*p* < 0.05) of PLT count than the arthritic control group. Modulation in hematological markers is shown in Table 3.

**TABLE 3:** Lawsone modulated Hematological Markers: Data is stated as mean ± SD (n = 06). **p < 0.01; ***p < 0.001 in contrast to negative control group and #*p* < 0.05; ##*p* < 0.01; ###*p* < 0.001 in correlation to positive control group.

Hematological parameters	Vehicle control group	Arthritic control group	Reference drug group	Lawsone 100 mg	Lawsone 200 mg
RBC (million/mm^3^)	9.865 ± 1.118	5.51 ± 0.846^*^	8.805 ± 1.061^**###**^	10.4 ± 1.2^**###**^	10.28 ± 0.667^**###**^
TLC (cells/μl)	7.067 ± 0.65	7.217 ± 0.595	6.933 ± 0.265	7.15 ± 0.689	7.267 ± 0.683
PLT count (10^9^/L)	782 ± 0.533	928.7 ± 40.06^**^	754.2 ± 50.9^**###**^	764.58 ± 0.818^**###**^	759.5 ± 57.89^**###**^
Hb (g/dL)	11.37 ± 1.133	8.89 ± 0.357^***^	11.12 ± 0.741^**###**^	11.57 ± 0.941^**###**^	11.68 ± 0.823^**###**^

### 3.8 Lawsone improved markers of liver function test

Significantly increased ALT (*p* < 0.001) and AST (*p* < 0.01) values were found in the arthritic control group than the vehicle control group. Hepatic parameters were found significantly improved in treated groups (Table 4).

**TABLE 4:** Lawsone improved markers of liver function test and showed no nephrotoxic effect: Values are stated as mean ± SD (n = 06). **p < 0.01; ***p < 0.001 in contrast to vehicle control and #*p* < 0.05; ##*p* < 0.01 in contrast to arthritic control group.

Biochemical parameters	Vehicle control group	Arthritic control group	Reference drug group	Lawsone 100 mg	Lawsone 200 mg
ALT (IU/L)	42 ± 3.098	54.83 ± 2.401^***^	43.5 ± 1.643^**###**^	43.17 ± 2.927^**###**^	43.67 ± 2.338^**###**^
AST (IU/L)	300.8 ± 39.0	400.8 ± 31.63^***^	332 ± 37.95^**#**^	315.7 ± 35.02^**##**^	335.5 ± 30.96^**#**^
Urea (mmol/L)	23.17 ± 4.491	24.5 ± 6.317	28 ± 6.261	30.5 ± 3.886	28.50 ± 3.782
Creatinine (mg/dL)	0.666 ± 0.051	0.666 ± 0.103	0.616 ± 0.116	0.633 ± 0.136	0.616 ± 0.04

### 3.9 No nephrotoxic effect of lawsone

The values of urea and creatinine, in all groups, were not statistically significantly different as compared to each other. This depicts that Lawsone is safe in terms of renal parameters (Table 4).

## 4 Discussion

In our current research, the anti-arthritic effect of Lawsone was analyzed, by using an FCA-induced rat arthritic model (Mobashar et al., 2022). FCA was chosen to induce arthritis in the Sprague-Dawley rat model because the clinical and pathological changes induced by FCA are considered similar to those that appear in human RA, which includes increased paw-edema volumes and thickening of the soft-tissue at the site of injection (Patil et al., 2011). It is the autoimmune disorder of connective tissues, especially the joints, cartilage, and bones, causing their disability and other systemic disorders, influenced by both genetic (MHC genes) and environmental factors (smoking, diet, obesity, infections, and microbiota (Croia et al., 2019). The major cause of RA development is the imbalance between the expression levels of pro-inflammatory and anti-inflammatory cytokines. Upregulation of pro-inflammatory cytokines or downregulation of anti-inflammatory cytokines results in the development of RA (Mobashar et al., 2020b). Characteristic features of RA involve the conversion of the synovial membrane into pannus, destroying the surrounding cartilage and bone with the presence of activated macrophages and synovial fibroblasts that form matrix metalloproteinase (Gautam, Singh, and Nainwani, 2013). Common complications in RA involve neurological disorders, tendon rupture, and joint damage (Yap et al., 2018). Its symptoms also involve fever, fatigue, and weight loss (Mobashar et al., 2020a). Chronic RA can also affect some major organs like the lung, heart, skin, eyes, kidneys, and digestive system (Radu and Bungau, 2021; Maity and Wairkar, 2022). Arthritis can cause severe disability, thus compromising health, and may even cause premature death (Murugananthan et al., 2013; Choudhary et al., 2014).

Lawsone has been shown to possess anti-inflammatory and anti-oxidant properties. These properties were accredited with ameliorative effect of lawsone in acute pancreatitis (Biradar and Veeresh, 2013). Lawsone is also known to inhibit paw edema in rat model by suppressing the inflammatory markers like, TNF-α and NF-ĸB (Vanco et al., 2017). In a recent study, we found Lawsone as the major active ingredient of *Eichhornia crassipes* and validated the plant for anti-arthritic property. It was suggested to evaluate the phytochemical for its potential against arthritis in future (Sattar et al., 2023). Therefore, we designed the current study to evaluate the anti-arthritic property of Lawsone using similar inflammatory markers and protocols as mentioned in our previous publication. Current research showed that the Lawsone downregulated the inflammatory markers, both *in vitro* and *in vivo*, and inhibited the development of RA in the treated groups. The anti-arthritic potential was evident by attenuation of histopathological parameters, arthritic score, paw edema, and hematological markers.

Cytokines play a crucial role in triggering the auto-immune system, leading to severe synovitis and the destruction of articular tissues (Alunno et al., 2017; Helli et al., 2019). The inhibitory effect of Lawsone on the expression of pro-inflammatory markers (TNF-α, IL-1β, IL-6, NF-ĸB, and VEGF) and matrix metalloproteinase enzymes (MMP-2 and MMP-3) was determined in current study.

Increased values of TNF-α result in inflammation and articular damage by stimulating the expression of cytokines and encouraging the immune cells such as, B-lymphocytes, T-lymphocytes, and NK cells, to proliferate and differentiate (Farrugia and Baron, 2016). It also acts synergistically with IL-1 in the degradation of proteoglycans, resulting in joint damage (Schiff, 2000). Inhibition of TNF-α results in the downregulation of the expression of other inflammatory cytokines (Wiens et al., 2010). IL-1β is responsible for bone erosion and pannus formation in RA, by binding to IL-1 receptor, mostly expressed in cartilage pannus (Harrison et al., 2008). It can stimulate pro-inflammatory cytokines in the cell lines significantly, especially the transcription and translation of MMPs, IL-6, and TNF-α (Chen et al., 2019).

IL-6 exerts its pleiotropic activity after binding to its receptor and triggering events via JAK-mediated events in target cells. It targets the plasmablasts and causes the proliferation of autoantibodies. Moreover, IL-6 actively promotes the maturation of T follicular helper cells, which then release IL-21, which is also a factor for the differentiation of B cells. Overall dysregulation of IL-6 is an important factor that leads to a decrease in inflammation.

NF-κB is found in inactive form in cytoplasm, where an inhibitory protein IкB is attached with it. Various cytokines such as, TNF-α, IL-1β, and chemokines phosphorylate IкB resulting in activaton of NF-κB (Roman-Blas and Jimenez, 2016). Activated NF-ĸB is notorious for mediating synovial inflammatory processes by proliferation of fibroblast-like synoviocytes, triggering transcription and expression of pro-inflammatory cytokines, causing the production of osteoclasts which leads to bone erosion, unwanted apoptosis, initiation of Th1 activation, and upregulation of synovial cells (Mobashar et al., 2020b; Ding et al., 2023).

VEGF is responsible for synovial inflammation, hyperplasia, and angiogenesis in the affected joints. It is produced by rheumatoid synoviocytes and upregulated by pro-inflammatory cytokines, such as TNF-α, IL-1, and IL-8, and hypoxia. It initiates a self-perpetuating cycle of the inflammatory response by attaching to Flt-1 receptors on macrophages, leading to synovial hyperplasia through various signaling intermediates such as, MAPK, Phosphoinositide 3-kinase (PI3K), protein kinase B (AKT), phospholipase C-γ (PlC-γ) and small GTPases (Yoo et al., 2008). Elevated serum levels of VEGF are detected in RA patients more than healthy controls or patients with osteoarthritis, thus VEGF is now taken as one of the first indicators in the investigation of RA (Malemud, 2007).

MMPs are also notorious for the bone remodeling, differentiation, recruitment and migration of osteoblasts and osteocytes, and solubilization of osteoid. TNF-α, IL-6, and IL-1β activate MMP genes in RA synovial fibroblasts through the binding of several different transcription factors (Araki and Mimura, 2017).

It is well known that cytokines, e.g., IL-1 and TNF-α, and cytokine inducers increase the levels of PGE2 during immune response (Davidson et al., 2001). PGE2 is a lipid-mediator and is responsible for bone erosion, destruction of articular cartilage, vasodilation, extravasation of fluid, and acute pain (Fattahi and Mirshafiey, 2012) in RA. It evokes cAMP/PKA signaling pathway through four different prostanoid E (EP) receptors on synovium, chondrocytes, liver, and monocytic phagocytes (McCoy, Wicks, and Audoly, 2002).

In current study, treatment with Lawsone downregulated the relative mRNA expressions levels of inflammatory markers, which are mostly known to be amplified in immunomodulatory diseases (Seibl et al., 2003; Huang et al., 2007; Kim et al., 2007; Gheorghe et al., 2009). The downregulation of these pro-inflammatory markers by Lawsone indicated that the amelioration of RA could be ascribed to the phytochemical’s potential immunomodulatory and anti-inflammatory properties (Izhar et al., 2021).

The most important function in the activation of proinflammatory cytokines and prognosis of RA is played by the Janus kinase/signal transducers and activators of transcription (JAK/STAT) signal transduction pathway. Normally, JAK/STAT negative regulators and active STAT protein inhibitors restrict cytokine activation. However, both regulators are malfunctioned in RA, resulting in constant positive signaling of JAK/STAT pathway and consequently increased expression levels of MMPs (Malemud, 2023). MMPs degrade the collagen matrix and promotes the inavasion of synovial panuus into the articular cartilage through various cell signaling pathways, including MAPK, NF-κB, and AMP-activated protein kinase (AMPK) pathwyas (Araki and Mimura, 2017). It is well known that imbalance between Th1 and Th2 cells is a key factor in the pathogenesis of RA, where Th1 mediated cell variants predominantly differentiate the others. This predominance is achieved by p38 MAPK pathway and inhitbion of p38 activity can prevent the differentiation of Th1 cells (Ding et al., 2023). The anti-inflammatory effect of Lawsone could possibly be mediated by mentioned pathways, however, further studies are required to evaluate the effects of Lawsone on signaling pathways involved in pathogenesis of RA.

In the current study, lab investigations showed reduced values of RBC count and Hb content in the positive control group, which indicates the anemic condition of rats (Song et al., 2013). It may be due to the deficiency in the generation of cells as a result of declined bone marrow functioning and/or reduced iron storage in the reticuloendothelial system. Anemia in RA is mainly due to the alteration in the production and functioning of hepcidin and ferroportin, due to pro-inflammatory cytokines, especially 1L-6. It can lead to other diseases as well such as arteriosclerosis (Atwa et al., 2022). Moreover, the rise in PLT counts in the positive control group indicates the immunomodulatory as a defense response to attacking pathogens (Naz et al., 2020). Treatment with Lawsone normalized the levels of evaluated hematological parameters (RBC, PLT, and Hb content), Likewise, evaluation of hepatic and renal markers also proved the safety of Lawsone in terms of hepatotoxicity or nephrotoxicity.

## 4 Conclusion

The current research proved that Lawsone possesses significant anti-inflammatory and anti-arthritic properties against FCA-induced RA in animal models. Treatment with Lawsone reduced arthritic progress and paw edema along with histopathological parameters. Diminution of RA may be attributed to the decline in the expression levels of inflammatory parameters like TNF- α, IL-1β, IL-6, NF-ĸB, MMP-2, MMP-3, and VEGF by the phytochemical. Lawsone also reduced the levels of PGE2 and results were found comparable to piroxicam in amelioration of RA. Current study presented the data on mRNA expression levels of inflammatory markers. For further validation of the data, it is suggested to analyse protein levels of tested inflammatory markers in future studies using Western Blot technique.

## Data Availability

The data used to support the findings of this study are available from the corresponding author upon reasonable request.

## Abbreviations

RA, Rheumatoid Arthritis; FCA, Freund’s Complete Adjuvant; H&E, Hematoxylin and Eosin; PGE2, Prostaglandin E2; ELISA, Enzyme-linked Immunosorbent Assay; ALT, Alanine Transaminase; AST, Aspartate Aminotransferase; Hb, Hemoglobin; CBC, Complete Blood Count; VEGF, Vascular Endothelial Growth Factor; TNF-α, Tumor Necrosis Factor- α; MMP-2 and MMP-3, matrix metalloproteinase; NF-κB, Nuclear Factor-Kappa B; IL-1β and IL-6, Interleukins.
